# Potentiation of the Effect of Thiazide Derivatives by Carbonic Anhydrase Inhibitors: Molecular Mechanisms and Potential Clinical Implications

**DOI:** 10.1371/journal.pone.0079327

**Published:** 2013-11-18

**Authors:** Kamyar Zahedi, Sharon Barone, Jie Xu, Manoocher Soleimani

**Affiliations:** Center on Genetics of Transport and the Department of Medicine, University of Cincinnati, Research Services, Veterans Affairs Medical Center, Cincinnati, Ohio; Aarhus University, Denmark

## Abstract

**Background:**

Carbonic anhydrase inhibitors (CAI) are mild diuretics, hence not widely used in fluid overloaded states. They are however the treatment of choice for certain non-kidney conditions. Thiazides, specific inhibitors of Na-Cl cotransport (NCC), are mild agents and the most widely used diuretics in the world for control of mild hypertension.

**Hypothesis:**

In addition to inhibiting the salt reabsorption in the proximal tubule, CAIs down-regulate pendrin, therefore leaving NCC as the major salt absorbing transporter in the distal nephron, and hence allowing for massive diuresis by the inhibitors of NCC in the setting of increased delivery of salt from the proximal tubule.

**Experimental Protocols and Results:**

Daily treatment of rats with acetazolamide (ACTZ), a known CAI, for 10 days caused mild diuresis whereas daily treatment with hydrochlorothiazide (HCTZ) for 4 days caused hardly any diuresis. However, treatment of rats that were pretreated with ACTZ for 6 days with a combination of ACTZ plus HCTZ for 4 additional days increased the urine output by greater than 2 fold (p<0.001, n = 5) compared to ACTZ-treated animals. Sodium excretion increased by 80% in the ACTZ plus HCTZ group and animals developed significant volume depletion, metabolic alkalosis and pre-renal failure. Molecular studies demonstrated ∼75% reduction in pendrin expression by ACTZ. The increased urine output in ACTZ/HCTZ treated rats was associated with a significant reduction in urine osmolality and reduced membrane localization of AQP-2 (aquaporin2).

**Conclusions:**

These results indicate that ACTZ down-regulates pendrin expression and leaves NCC as the major salt absorbing transporter in the distal nephron in the setting of increased delivery of salt from the proximal tubule. Despite being considered mild agents individually, we propose that the combination of ACTZ and HCTZ is a powerful diuretic regimen.

## Introduction

Kidney plays an essential role in vascular volume homeostasis through the reabsorption of filtered sodium, chloride and water in various nephron segments. The proximal tubule reabsorbs around 60% while the thick ascending limb reabsorbs almost 30% of filtered load of NaCl [1]–[6]. The distal convoluted tubule (DCT) reabsorbs approximately 7–9% of the filtered salt and the remaining fraction is reabsorbed in the connecting tubule (CNT) and the collecting duct (CD) [7]–[9].

The apical Na-Cl co-transporter (NCC) is the main salt absorbing transporter in the DCT and is specifically inhibited by thiazide derivatives [7], [8]. The Cl^−^/HCO_3_^−^ exchanger pendrin [10] works in tandem with the epithelial sodium channel ENaC and in part with the sodium dependent Cl^−^/HCO_3_ exchanger (NDCBE) to mediate salt reabsorption in CNT and CCD [9], [11].

Carbonic anhydrases play important roles in acid base transport in the proximal tubule and the collecting duct [12], [13]. Inhibition of carbonic anhydrase activity in the proximal tubule by acetazolamide blocks the apical Na^+^/H^+^ exchanger activity and decreases sodium and bicarbonate reabsorption [12]–[14]. Short-term (1 or 2 weeks) inhibition of carbonic anhydrases causes significant remodeling of cellular profile in the collecting duct, with a specific reduction in B-intercalated cells [15]. Carbonic anhydrase inhibitors are regularly used for the treatment of elevated intracranial pressure in pseudotumor cerebri and increased intraocular pressure in glaucoma by reducing the production of cerebrospinal fluid (CSF) and aqueous humor, respectively [16], [17].

Hydrochlorothiazide (HCTZ) is the most widely used diuretic in the world for the treatment of mild and moderate hypertension [18], [19]. Despite being a specific inhibitor of NCC in the DCT hydrochlorothiazide causes a very mild diuretic response [18]–[20]. This observation is in agreement with studies indicating that NCC deletion in mouse causes very little salt wasting under basal conditions [21]. A recent study by our laboratory tested the hypothesis that NCC and pendrin, which are located in close proximity of each other in the distal nephron, compensate for loss of the other under basal conditions thereby masking the role that each plays in salt reabsorption [22]. Toward this goal, pendrin and the NaCl co-transporter (NCC) double-knockout mice were generated, which showed significant salt and fluid wasting along with volume depletion and pre-renal failure under baseline conditions [22].

We hypothesize that carbonic anhydrase inhibition by ACTZ down-regulates pendrin, therefore leaving NCC as the only major salt absorbing transporter in the distal nephron. As such, we postulate that the addition of HCTZ, which inhibits NCC, should cause profound diuresis, subsequent to the inactivation of pendrin and NCC in the face of increased delivery of salt from proximal tubule. The results presented in this manuscript support this hypothesis. We propose that patients that are treated with ACTZ for pseudotumor cerebri (idiopathic intracranial hypertension) or other non-kidney conditions, such as glaucoma, should avoid taking HCTZ for hypertension due to profound diuretic effect of the combination therapy.

## Results

### Effect of ACTZ, HCTZ or ACTZ plus HCTZ on kidney function parameters

**Figure 1:**
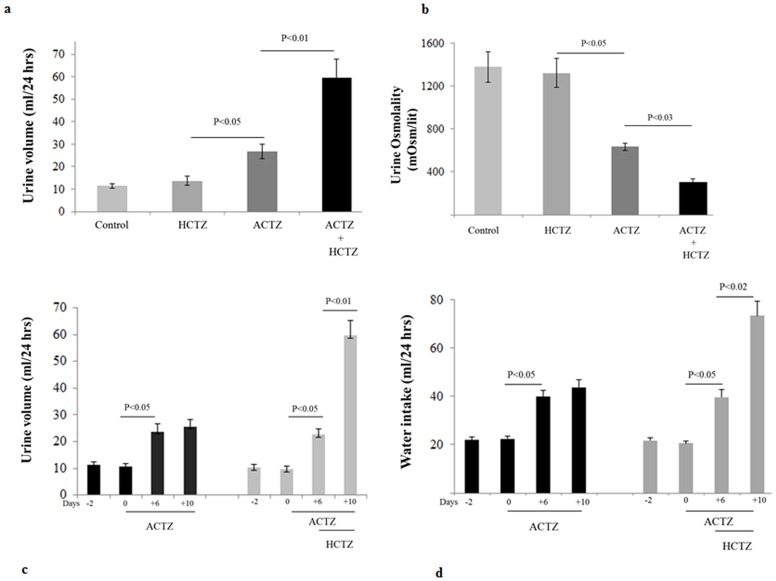
Effect of acetazolamide, hydrochlorothiazide, and acetazolamide plus hydrochlorothiazide treatment on kidney function parameters. Rats (n = 5/group) treated with vehicle (10 days), ACTZ (10 days), HCTZ (4 days) and pretreatment with ACTZ (6 days) followed by treatment with ACTZ/HCTZ for 4 days (10 days total) were subjected to balanced studies. **a. Urine output.** Urine output was 11.5 ml in vehicle treated, and 13.7, 25.5 and 59 ml/24 hour in HCTZ, ACTZ and ACTZ + HCTZ-treated animals, respectively. **b. Urine osmolality.** Urine osmolality was >1300 mOsm/lit in vehicle treated, and decreased to ∼630 in ACTZ and to ∼320 mOsm/lit in ACTZ + HCTZ-treated groups. **c. Time course of daily urine output before and after treatment with ACTZ and HCTZ.** Urine output is shown for animals treated with either ACTZ for 10 days or ACTZ for 6 days followed by ACTZ plus HCTZ for 4 more days. **d. Time course of daily water intake of vehicle-, ACTZ- or ACTZ/HCTZ-treated rats.** Water intake increased significantly with the administration of ACTZ (P<0.05) and again with the administration of HCTZ in conjunction with ACTZ (P<0.02).

**Figure 1a** (top panel) examines the effect of ACTZ and HCTZ, alone or in combination on kidney function parameters. Daily treatment with ACTZ causes moderate diuresis in rats, with urine output increasing from 11.5 to ∼24 ml/day (data shown for day 10 of ACTZ treatment; p<0.05, n = 5). Daily injection with HCTZ alone for 4 days caused a very mild diuresis, with urine output increasing from a baseline of 11.0 to 13.7 ml/day on day 4 of treatment (data shown for day 4 of HCTZ treatment; p>0.05, n = 5). However, in rats that were primed with ACTZ for 6 days, daily treatment with HCTZ and ACTZ for 4 additional days increased the urine output from 25 to 59 ml/day on the final day of HCTZ/ACTZ co-administration (data shown for day 4 of HCTZ/ACTZ co-treatment, p<0.001, n = 5). Corrected for body weights the urine outputs are 54.3+/−4.4, 66.6+/−8.50, 105.8+/−9.8 and 242.8+/−28.62 ml/kg/day for control, ACTZ, HCTZ and ACTZ/HCTZ treated groups, respectively, with ACTZ/HCTZ treated rats showing significant differences with the 3 other groups (p<0.004 vs. all three other groups, n = 5 in each group). **Figure 1b** shows urine osmolality measurements in experimental groups and indicates that while HCTZ treatment for 4 days had no significant effect on urine osmolality, ACTZ-treated rats showed a reduction in their urine osmolality after 6 and 10 days of treatment (p<0.05 vs. vehicle; n = 5/group). The highest reduction in urine osmolality, however, belonged to ACTZ/HCTZ-treated group (p<0.01 vs. vehicle and <0.05 vs. ACTZ alone; n = 5/group).

The greater than 2 fold increase in urine output by HCTZ/ACTZ group compared to HCTZ treated animals (Fig. 1a) warranted the examination of time matched control vs. ACTZ alone. Toward this end, the effect ACTZ, alone or in combination with HCTZ, on urine output was determined. As shown in Fig. 1c and d, co-treatment with HCTZ and ACTZ for 4 days in ACTZ-pretreated animals significantly increased urine output (**Fig. 1c**) and water intake (**Fig. 1d**). The continuation of ACTZ treatment alone for 10 days did not increase the urine output beyond that of the 6 days of ACTZ treatment (**Fig. 1c**).

### Effect of ACTZ plus HCTZ on salt excretion, vascular volume and kidney function

**Figure 2:**
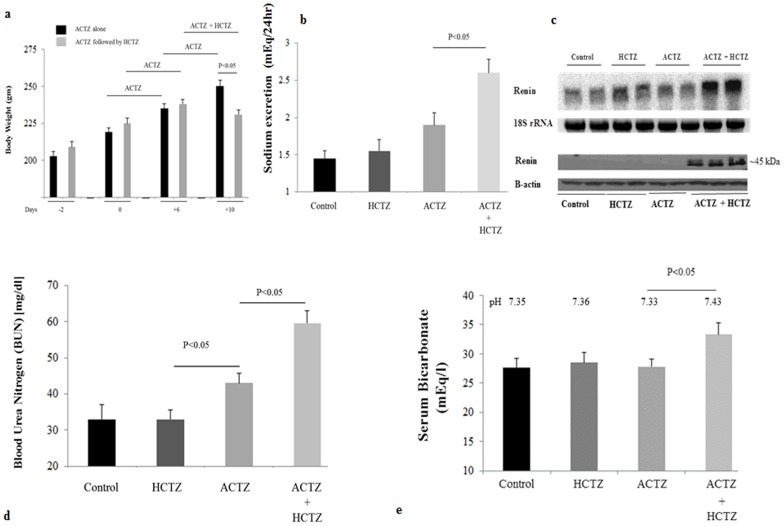
Effect of acetazolamide and hydrochlorothiazide on vascular volume status. Rats (n = 5/group) treated with vehicle (10 days), ACTZ (10 days), HCTZ (4 days), and pretreatment with ACTZ (6 days) and then treated with ACTZ/HCTZ for 4 days (10 days total) were subjected to balanced studies. Urinary sodium levels were determined for urine samples collected on the final day of the study for each group. Animals were euthanized. Blood samples and kidneys and were collected from each animal. Serum samples were analyzed for blood urea nitrogen. **a. Body weights.** Daily weight monitoring in animals before and after the initiation of ACTZ treatment with or without HCTZ indicates that rats treated with ACTZ plus HCTZ lost weight compared to rats on ACTZ alone. ACTZ treated animals (no HCTZ) showed comparable body weight gain to vehicle treated animals (Results). **b. Salt excretion.** Sodium excretion was ∼1.45 mEq/24 hrs in vehicle treated, 1.9 mEq/24 hrs in ACTZ and 2.6 mEq/24 hrs in ACTZ + HCTZ-treated groups. * indicates significant difference (see text for more information). **c. Expression of renin in kidneys of Vehicle-, HCTZ-, ACTZ- and ACTZ/HCTZ-treated animals.** The mRNA expression and protein levels of renin (left and right panels) were measured by Northern Hybridization and Western Blot in kidneys of control and animals that were treated with ACTZ, HCTZ or ACTZ plus HCTZ. Results shown are representative of 2 separate studies examining 4 different rats from each treatment group. **d. BUN levels in Vehicle-, HCTZ-, ACTZ- and ACTZ/HCTZ-treated animals.** BUN concentration levels were measured in animals treated with ACTZ, HCTZ or both. **e. Systemic acid base parameters.** Animals treated with ACTZ + HCTZ have significantly elevated arterial blood bicarbonate levels and pH compared to other experimental groups.

**Figure 2a** depicts body weight measurements in ACTZ-treated vs. ACTZ/HCTZ-treated animals. As indicated, ACTZ-treated animals continued to gain weight throughout the study. However, ACTZ-primed animals that were co-treated with ACTZ and HCTZ for 4 additional days showed a significant reduction in body weight compared to animals treated with ACTZ alone for the same duration. Treatment with HCTZ alone for 4 days did not adversely affect the weight gain compared to vehicle-treated rats (both groups showed normal daily weight gain of 4–4.7 g). Food intake in ACTZ/HCTZ treated group was comparable to that of ACTZ-treated group (0.72+/−0.03 vs. 0.75+/−0.02 g food/gram body weight/day, p>0.05), indicating that the weight loss in ACTZ/HCTZ-treated group reflected the loss of fluid through kidneys.

**Figure 2b** depicts urine sodium concentration in various experimental groups and indicates that sodium excretion increased by 37% in ACTZ treated and by 80% in ACTZ/HCTZ treated animals compared to the control group. HCTZ treatment had no significant effect on sodium excretion.

To determine whether increased salt excretion and fluid loss causes volume depletion, the expression of renin in kidneys of experimental groups was measured. Northern and western blot analyses indicate that compared to other treatment groups the ACTZ/HCTZ treated animals had the highest expression levels of renin (**Figure 2c** **, top and bottom panels**).

To ascertain the effect of vascular volume depletion on kidney function, blood urea nitrogen (BUN) and creatinine levels were measured in animals from the 4 treatment groups (n = 5/group). **Figure 2d** indicates that BUN levels of ACTZ-treated rats are slightly increased compared to that of vehicle-treated animals. The ACTZ/HCTZ-treated rats showed a significant increase in their BUN levels compared to ACTZ- and vehicle-treated rats, consistent with pre-renal failure. Serum creatinine levels also increased significantly in ACTZ/HCTZ-treated group compared to other experimental groups.

Arterial blood gas analysis indicates that HCTZ, ACTZ and vehicle treated animals had similar acid base profiles (**Figure 2e**). The ACTZ/HCTZ-treated group, however, showed significant elevation in serum bicarbonate and arterial pH, consistent with the development of metabolic alkalosis (**Figure 2e**). Serum electrolyte profile showed low serum sodium but normal potassium levels in ACTZ/HCTZ-treated animals (**Table 1**).

**Table 1:** Serum electrolyte profile.

	Control	HCTZ	ACTZ	ACTZ + HCTZ
[Na^+^] (mEq/l)	139+/−1	138+/−1	139+/−1	133+/−2
[K^+^] (mEq/l)	4.6+/−0.2	4.5+/−1	4.4+/−0.2	4.0+/−0.3

Serum sodium and potassium levels in experimental animals (n = 5/group) are shown.

### Acetazolamide (ACTZ) down-regulates pendrin

**Figure 3:**
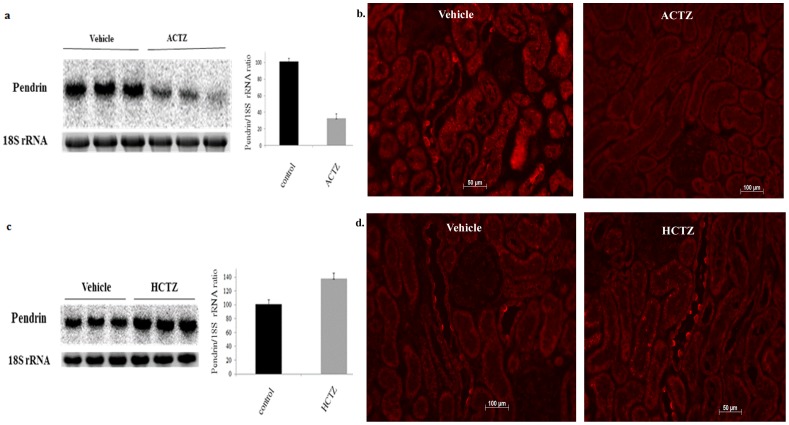
Effect of acetazolamide on pendrin expression in rat kidney. The expression of pendrin and a. Expression of pendrin mRNA in animals treated with ACTZ. Expression of pendrin in animals treated with ACTZ for 6(lower panel). Results shown are representative of 2 separate studies examining 5 different rats from each treatment group. **b. Pendrin expression in kidneys of rats treated with ACTZ.** Left panels depict immunofluorescent labeling of kidneys from control rats with pendrin antibodies and show multiple pendrin positive cells in cortical collecting ducts. Right panels show kidneys from ACTZ treated animals nd stained with pendrin antibody. **c. mRNA expression of pendrin in kidneys of animals treated with HCTZ.** Expression of pendrin in animals treated with HCTZ for 4 days is shown. The 18S rRNA levels confirm the loading equality of RNA samples (lower panel). Results shown are representative of 2 separate studies examining 5 different rats from each treatment group. **d. Pendrin expression in kidneys of rats treated with HCTZ.** Pendrin immunofluorescent labeling in kidneys from control rats (right panels) and rats treated with HCTZ (right panel).

**Figure 3** shows the expression of pendrin in kidneys of rats treated with acetazolamide for 6 days. The expression of pendrin, as determined by northern blot analysis and immuneofluorescent microscopic analysis, was down regulated in ACTZ- compared to vehicle-treated animals (**Fig. 3a and 3b**). Treatment of rats with HCTZ for 4 days increased the expression of pendrin mRNA (**Fig. 3c**), with no apparent increase in protein abundance as determined by immunofluorescence labeling studies (**Fig. 3d**).

**Figure 4:**
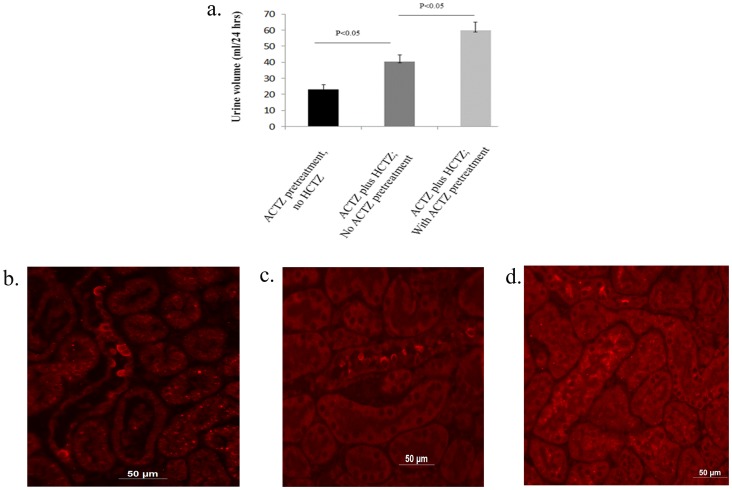
Effect of simultaneous treatment of ACTZ and HCTZ with no ACTZ pretreatment on diuresis and pendrin expression. **a.** The magnitude of diuresis in rats co-treated with ACTZ and HCTZ but without ACTZ pretreatment was significantly less than in rats pretreated with ACTZ followed by ACTZ and HCTZ co-treatment. **b, c and d.** Immunofluorescent microscopic analysis of pendrin in control (b), simultaneous treatment with ACTZ and HCTZ (c) and sequential treatment with ACTZ and HCTZ (d).

The role of ACTZ pretreatment on the magnitude of diuresis in the ACTZ/HCTZ co-treatment group (**Fig. 1**) was examined by the following experiments: In group 1, rats (n = 5) were simultaneously treated with ACTZ and HCTZ for 2 days with no ACTZ pretreatment (no time for pendrin down-regulation by ACTZ) whereas the rats in group 2 (n = 5) received ACTZ pretreatment for 6 days followed by 2 days of ACTZ/HCTZ co-treatment. **Figure 4a** shows that the urine output in group 1 (simultaneously treated rats with no ACTZ pretreatment) were significantly lower than that of group 2 (pretreated with ACTZ followed with ACTZ/HCTZ co-treatment). Urine sodium levels in simultaneously-treated rats were significantly less than those in rats sequentially treated with ACTZ and HCTZ (1.55+/−0.05 vs 1.86+/−0.05 mEq/24 hrs, p<0.03, n = 5). The expression of pendrin was significantly higher in simultaneously treated vs. that of sequentially treated animals (**Fig. 4c** **, vs.** **Fig. 4d**). **Figure 4b** is an image from a control (vehicle-treated) animal. Taken together, these results indicate that the maximum synergistic effect of HCTZ and ACTZ on salt excretion requires pretreatment with ACTZ (which is associated with the down regulation of pendrin).

### Regulation of AQP2 in response to ACTZ/HCTZ treatment

**Figure 5:**
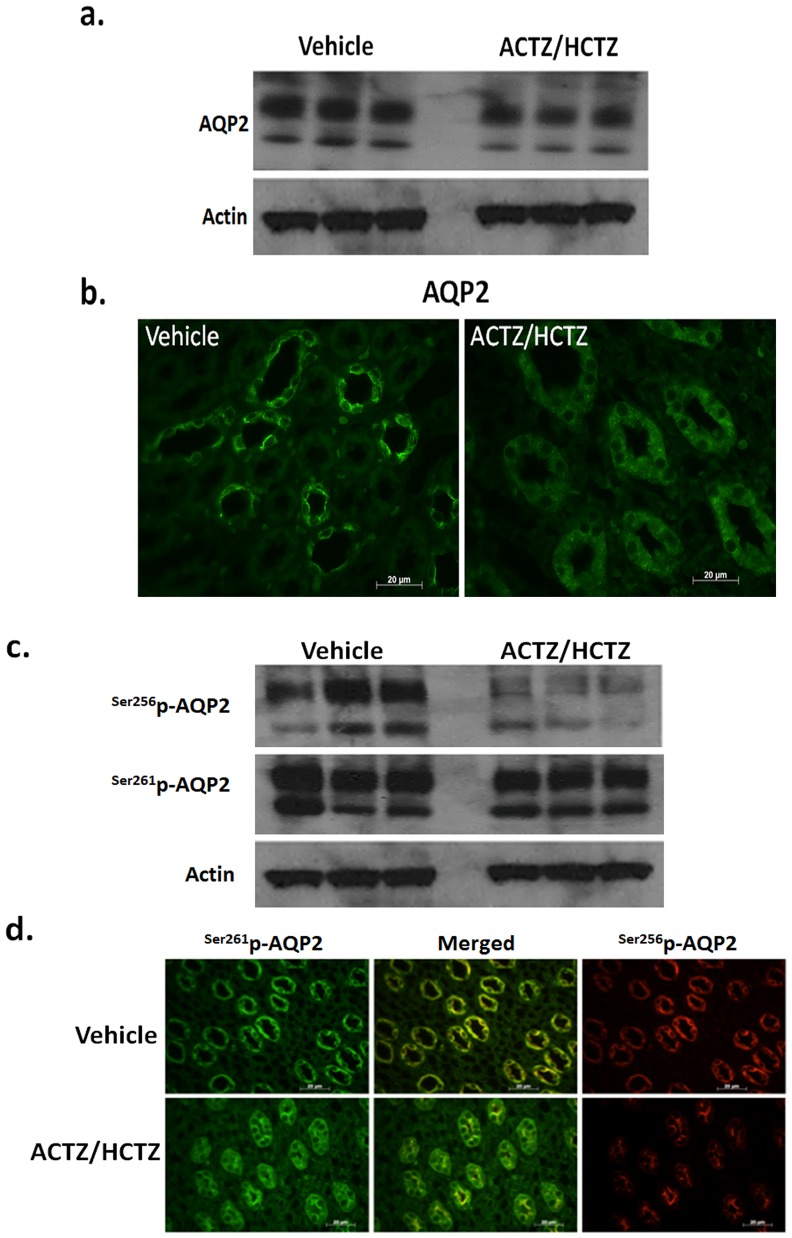
Effect of ACTZ/HCTZ treatment on AQP2 expression and trafficking. a. Western blot analysis of kidney extracts from the vehicle and ACTZ/HCTZ treated animals revealed that the expression of total AQP2 was significantly reduced in the kidneys of the latter. Results shown are representative of 2 separate studies examining 5 different rats from each treatment group. b. Immunofluorescent microscopic examination of AQP2 expression in the kidneys of vehicle and ACTZ/HCTZ treated animals indicates that its expression is reduced in the latter group. c. Comparison of the phosphorylated AQP2 revealed that compared to vehicle treated animals the cellular content of ^Ser256^p-AQP2 were significantly reduced while those of ^Ser261^p-AQP2 were only marginally affected in the ACTZ/HCTZ treated animals. Results shown are representative of 2 separate studies examining 5 different rats from each treatment group. d. Immunofluorescent microscopic examination of ^Ser256^p-AQP2 and ^Ser261^p-AQP2 in the kidneys of vehicle and ACTZ/HCTZ treated animals indicates the cellular levels of both phosphorylated proteins are reduced in the latter. In addition these studies indicate that although there is substantial co-staining for both phosphotypes, ^Ser256^p-AQP2 is mainly surface bound while ^Ser261^p-AQP2 is primarily intracellular.

The significant increase in urine output of ACTZ/HCTZ treated groups was associated with a reciprocal reduction in urine osmolality (**Fig. 1**) despite the fact that these animals are volume depleted, as shown by increased expression of kidney renin and elevation of BUN (**Fig. 2**). The impaired urine concentration in the context of volume depletion is a surprising findings and points to impaired water reabsorption (reflecting compromised AQP2 expression and/or function). In the next series of experiments we examined the expression of AQP2 in control and ACTZ/HCTZ-treated animals. Accordingly, the abundance of AQP2, ^Ser256^p-AQP2 and ^Ser261^p-AQP2 were assessed. Western blot analysis of kidney extracts from the vehicle- and ACTZ/HCTZ-treated animals revealed that the expression of total AQP2 was reduced in the kidneys of the latter (**Fig. 5a**). These results were confirmed by immunofluorescent examination of kidneys from vehicle- and ACTZ/HCTZ-treated animals (**Fig. 5b**). Additionally, the levels of ^Ser256^p-AQP2 were reduced while the levels of ^Ser261^p-AQP2 were mildly affected in the ACTZ/HCTZ treated animals (**Fig. 5c**). The results of our immunofluorescent microscopy studies indicate that ACTZ/HCTZ treated animals have reduced levels of total and membrane bound/cycling AQP2 (^Ser256^p-AQP2, ^Ser256–261^p-AQP2) while their intracellular/cycling AQP2 (^261^p-AQP2) levels are elevated (**Fig. 5d**).

## Discussion

The use of diuretics in fluid overloaded states is determined by their strength, mechanism of action and possible side effects. Loop diuretics, which inhibit the apical Na-K-2Cl co-transporter (NKCC2) in the thick limb, are powerful natriuretic agents but have numerous side effects, including severe hypokalemia [5]–[7]. Thiazides are the most widely used diuretic for mild hypertension and as a combination therapy for moderate hypertension [18). They have also been used in conjunction with loop diuretics for treatment of severe fluid overload [18], [23].

Carbonic anhydrase (CA) inhibitors are known to inhibit the sodium and bicarbonate reabsorption in the proximal tubule through the inhibition of CA-2 and CA-4 and impair the collecting duct acid secretion, which is predominantly mediated via H^+^-ATPase in A-intercalated cells, through the inhibition of CA-2 [11]–[14]. They are considered mild diuretics and therefore not commonly used for the treatment of fluid overloaded states. Mice deficient in CA-2 show a significant reduction in the number of intercalated cells, along with the down-regulation of pendrin [24]. These results are in agreement with our studies in rats treated with acetazolamide, and indicate the importance of CA-2 in the development and remodeling of intercalated cells.

Recently, we demonstrated that the simultaneous knockout of pendrin and NCC genes in mice causes massive salt wasting and volume depletion, despite the fact that deficiency of either pendrin or NCC in isolation does not result in any noticeable salt wasting under basal conditions [22]. Given the up-regulation of pendrin in kidneys of NCC KO mice [26], these results indicate that pendrin plays an important role in compensatory salt absorption in kidneys of NCC KO mice [22]. The results of the current investigation extend the latter observations and demonstrate that as a result of pendrin down-regulation subsequent to carbonic anhydrase inhibition, NCC becomes the major salt absorbing transporter in the distal nephron in the setting of increased delivery of salt from proximal tubule.

Additionally, the urine osmolality is reduced while the renin expression is elevated in HCTZ/ACTZ treated rats (Figs. 2, 3). These observations suggest that the water salvage mechanism in the kidneys of animals treated with ACTZ/HCTZ is severely impaired. Our results suggest that the inability to conserve water in ACTZ/HCTZ treated animals in the face of dehydration is primarily due to reduced expression of AQP2 and its diminished surface expression as evidenced by a decrease in the abundance of ^Ser256^p-AQP2 (Fig. 5). Our recent studies in pendrin/NCC double KO mice show remarkable phenotypic similarity to the current HCTZ/ACTZ-treated rats with respect to their impaired ability to concentrate the urine [27]. In those studies we demonstrate that pendrin/NCC dKO mice have nephrogenic DI and decreased ^Ser256^p-AQP2 levels in medullary collecting ducts [27]. Taken together, these studies indicate that severe salt wasting might induce signaling (i.e. through PGE2 receptor) that counteracts the effect of V2 receptor activation and differentially affects AQP2 phosphorylation. Whether the impairment in ADH effect in ACTZ/HCTZ-treated rats might be in part due to the presence of metabolic alkalosis [28] remains speculative.

Aside from being mild diuretics, carbonic anhydrase inhibitors such as acetazolamide are the treatment of choice for certain non-kidney conditions. These include glaucoma, pseudotumor cerebri (increased intracranial pressure, IIP), and mountain sickness [16], [17], [25]. Carbonic anhydrase inhibitors reduce intraocular pressure and intracranial pressure by reducing the production of vitreous humor and cerebrospinal fluid (CSF), respectively [16], [17]. They also improve hypoxia in mountain sickness [25]. We propose that patients that are on acetazolamide analogs for non-kidney condition such as IIH or glaucoma should avoid taking thiazide derivatives for hypertension due to the strong possibility of developing massive volume depletion subsequent to profound diuresis and salt wasting. Furthermore, individuals who are on thiazides for the treatment of hypertension should switch to other classes of hypertensive medications if they need to start acetazolamide for prevention of mountain sickness or treatment of glaucoma. The reason for this warning is that these patients usually have normal vascular volume and are not fluid overloaded. As a result, severe diuresis in these patients could cause volume depletion, hypotension and possibly renal failure.

These are the first studies to demonstrate that acetazolamide and hydrochlorothiazide, which are historically known to be mild diuretics, can function as a powerful diuretic regimen when administered together. Preliminary studies from our on-going studies in humans (n = 5) with nephrotic syndrome and preserved kidney function (GFR >60 ml/min) demonstrated that 6 days of pretreatment with acetazolamide followed by 6 days of co-treatment with acetazolamide and hydrochlorothiazide caused more than 5% weight loss and improvement in peripheral edema (P<0.05 vs. before therapy; personal observation), supporting the notion that this combination is a strong and effective diuretic regimen. One has to be cautious with extrapolating from these studies to other models of fluid overload, such as congestive heart failure.

The current studies strongly suggest that the primary reason for mild diuresis by acetazolamide is the compensatory activation of NCC subsequent to increased delivery of salt from the proximal tubule, therefore blunting the expected salt wasting. Equally important to NCC up-regulation in ACTZ-treated rats is the down-regulation of pendrin, which leaves DCT and CCD with little compensatory salt absorbing mechanisms when NCC is inhibited.

The absence of hypokalemia in ACTZ/ACTZ-treated animals (Table 1), despite severe volume depletion, increased renin/aldosterone pathway and metabolic alkalosis is intriguing. One plausible explanation could be that pendrin works in conjunction with the sodium channel (ENaC) to absorb NaCl, and the inhibition of pendrin would blunt the activity of ENaC, therefore impairing the secretion of potassium via K+ channels in exchange for sodium absorption through ENaC [29]. Alternatively, the down regulation of pendrin by acetazolamide could render the sodium-dependent Cl^−^/HCO_3_^−^ exchanger (NDCBE) inactive in B-intercalated cells [11], [30] which can result in enhanced salt excretion with no potassium wasting (Table 1).

One interesting finding in current studies was the presence of metabolic alkalosis in rats co-treated with ACTZ and HCTZ (Fig. 2). Given the ability of CAs to facilitate bicarbonate reabsorption in the proximal tubule [12], [14], the anticipation is that CAIs will cause bicarbonate wasting and result in proximal renal tubular acidosis (RTA). Published reports, however, indicate that rats treated with ACTZ for 7 days or 14 days do not develop proximal RTA [15]. The presence of metabolic alkalosis in ACTZ/HCTZ co-treated rats (Fig. 2) might reflect the impact of pendrin downregulation in the context of volume depletion. Pendrin deficient mice, but not their wild type littermates, develop metabolic alkalosis when subjected to salt-depleted diet [31], indicating the important role of pendrin-mediated bicarbonate secretion and chloride reabsorption in vascular volume homeostasis and acid base balance during volume depletion. Similarly, it is highly plausible that the downregulation of pendrin in ACTZ/HCTZ co-treated rats might play an important role in the generation of metabolic alkalosis in these animals. Whether the ACTZ-mediated RTA in human [12], [14] and its absence in rat [15] reflect species differences remains speculative.

One of the complications of carbonic anhydrase inhibitors is enhanced calcium excretion, which can result in nephrocalcinosis [32], [33]. This is the opposite of the effect of HCTZ, which is known to decrease calcium excretion [34], [35]. It is intriguing that rats treated with ACTZ and HCTZ did not show any increment in calcium excretion, indicating that the hypocalciuric effect of HCTZ can offset the hypercalciuric effect of ACTZ.

**Figure 6:**
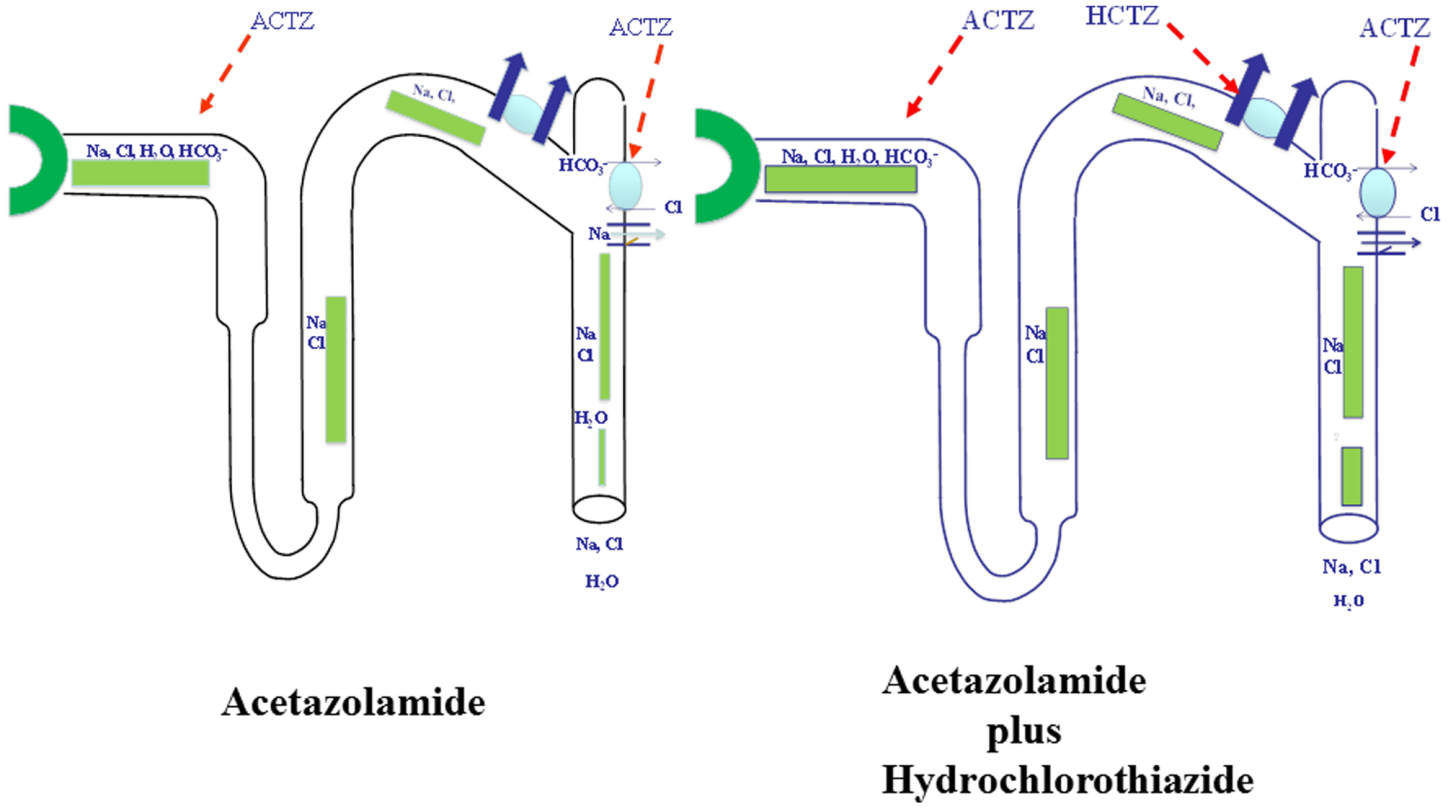
A proposed schematic diagram depicting the synergistic diuretic effects of ACTZ and HCTZ in rats. The combined inhibition of NCC and pendrin can cause significant salt and water wasting in the setting of increased delivery of salt from proximal tubule. Figure 6 (left panel): The carbonic anhydrase inhibitor Acetazolamide (ACTZ) increases the delivery of salt out of proximal tubule, down-regulates pendrin and activates NCC, which is depicted with thick yellow arrows; Fig. 6 (right panel): Combined ACTZ and HCTZ treatment in rats pretreated with ACTZ causes severe salt wasting due to the inhibition of NCC in the setting of pendrin downregulation and increased delivery of salt from the proximal tubule. The increased salt excretion in the latter group (Fig. 6b) is depicted with wide diameter light green columns in the collecting duct.

In conclusion, ACTZ down regulates pendrin, leaving the thiazide sensitive transporter NCC, as the major salt absorbing transporter in the distal nephron in the setting of increased delivery of salt from proximal tubule. **Figure 6, left panel** is a schematic diagram indicating that the activation of NCC and the downregulation of pendrin in the context of increased delivery of salt from the proximal tubule by acetazolamide (**panel 2**). **Figure 6, right panel**, depicts the impact of hydrochlorothiazide (an inhibitor of NCC) on salt excretion in the presence of acetazolamide.

## Materials and Methods

### Animal models and balanced studies

Sprague Dawley rats (150–200 gm) were placed in metabolic cages and divided into four different groups: 1) Control, 2) HCTZ treatment (40 mg/kg BW; [36]) for 4 days, 3) ACTZ treatment (100 mg/kg BW; [37]) for 10 days, and 4) ACTZ treatment for 6 days followed by ACTZ plus HCTZ for 4 more days (a total of 10 days of ACTZ and 4 days of HCTZ). Details of generation of the thiazide sensitive cotransporter (NCC) null mice have been reported elsewhere [21]. Balanced studies (water intake, urine output, food intake, body weight and urine osmolality) were measured daily, and at the end of the experiments animals were euthanized and their blood and kidneys were collected.

Animals were housed and cared for in accordance with the Institutional Animal Care and Use Committee (IACUC) at the University of Cincinnati. All animal handlers were IACUC-trained. Animals had access to food and water *ad libitum*, were housed in humidity, temperature, and light/dark controlled rooms, and were inspected daily. Animals were euthanized with the use of excess anesthetics (pentobarbital sodium) according to institutional guidelines and approved protocols. All animal studies described in this manuscript were approved by the University of Cincinnati IACUC Review Board.

### Immunofluorescence labeling

Animals were euthanized with an overdose of pentobarbital sodium, and kidneys were removed, cut in tissue blocks, and fixed in formaldehyde solution overnight at 4°C. The tissue was transferred to 70% ethanol and embedded in paraffin, and 5-μm sections were cut and stored until used. Single-immunofluorescence labeling using anti-pendrin [10], [38] or AQP2 antibodies was performed as previously described [39]–[41]. Immunofluorescent double labeling using rabbit anti ^Ser256^p-AQP2 and ^Ser261^p-AQP2 antibodies was performed as follows. After antigen retrieval with sodium citrate, sections were incubated overnight at 4 degree with the first polyclonal primary antibody, washed with PBS and incubated in Alexa Fluor goat-anti-rabbit 594 IgG (H+L) (Invitrogen, Carlsbad, CA) for one hour at room temperature. Following the PBS washes, the sections were incubated in 10% normal rabbit serum and then incubated in a Fab goat-anti-rabbit IgG (H+L) (Jackson ImmunoResearch, West Grove, PA). The second polyclonal primary antibody was added to the sections for an overnight incubation at 4 degrees. Sections were washed in PBS and placed in Alexa Fluor goat-anti-rabbit 488 IgG (H+L) (Invitrogen, Carlsbad, CA) for one hour at room temperature. Sections were washed in PBS, dried and cover slips were applies using Vectashield Mounting Medium (Vector Labs, Burlingame, CA).

### Western blotting

For Western blot analysis, membrane proteins isolated from rat kidneys were size-fractionated by SDS/PAGE (90 μg/lane), transferred to nitrocellulose membrane. The blots were examined for the expression of renin, AQP2, ^Ser256^p-AQP2, ^Ser261^p-AQP2 and Actin. The secondary antibodies were anti-sheep IgG conjugated to horseradish peroxidase for renin, anti-goat IgG conjugated to horseradish peroxidase for AQP2 and actin and anti-rabbit IgG conjugated to horseradish peroxidase for ^Ser256^p-AQP2 and ^Ser261^p-AQP2. The bands were visualized using the HRP Chemiluminescence Kit (Invitrogen, Carlsbad, CA) and images were captured on light-sensitive imaging film (MidSci, St. Louis, MO).

### Antibodies

Pendrin antibodies were generated in our laboratory as described (10). The monoclonal antibody against AQP2 (39) was kindly provided by Dr. Ann Blanchard (Dr. Anne Blanchard, Centre d'Investigations Cliniques, Hôpital Européen Georges Pompidou, Paris, France). Antibodies against renin (MyBiosource, #MBS315812, San Diego, CA), Actin and AQP2 (Santa Cruz Biotechnology, Santa Cruz, CA), ^Ser256^p-AQP2 (Assay Biotech, Sunnyvale, CA) and ^Ser261^p-AQP2 (Taipei, Taiwan) were purchased from vendors.

### Northern Blot analysis

Northern analyses were performed according to established protocols [42]. Briefly, total cellular RNA was extracted from kidneys of rats, quantitated spectrophotometrically, and stored at −80°C. Total RNA samples (30 μg/lane) were fractionated on a 1.2% (g/dl) agarose-formaldehyde gel, transferred to Magna NT nylon membranes, cross-linked by UV light, and baked. Hybridization was performed according to established methods. The membranes were washed, blotted dry and exposed to a PhosphorImager screeen. The signal strength of hybridization bands was quantitated by densitometry using ImageQuaNT software.

Gene specific, PCR amplified rat cDNA fragments were used as specific probes for pendrin and NCC. The sequences of primers for NCC probe were GTGATCCTCTACCTGCGACTC (sense) and TTGTCTTCAGATGCTGGGATC (antisense). The fragment spans the nucleotides 481 to 959 of NCC cDNA (Genebank # NM_001205311). The sequences of primers for Pendrin probe were GTGTTTGGAGGTTTGCAGATTG (sence) and TCACAATCACAGCTGAGATGAG (antisence). The fragment spans the nucleotides 819 to 1509 of Pendrin cDNA (Genebank # NM_011867). For renin, a PCR fragment encoding nucleotides 291 to 600 (accession number NM_031192) was used. Additionally, probes for sodium channel subunits α (nucleotides 1197 to 1890), β (nucleotides 1012 to 1848) and γ (nucleotides 135 t0 790), as well as AQP2 (nucleotides 102 to 397) and NDCBE (nucleotides 500 to 919 of mouse cDNA, accession number NM_021530) were used.

### Blood chemistry and electrolyte analysis

Serum concentration of Na^+^, K^+^, and HCO_3_^−^ were quantified in the blood using an i-STAT-1 analyzer with i-STAT EG7+ cartridges. Blood urea nitrogen (BUN) levels were quantified with a BUN assay kit.

### Urine osmolality and sodium excretion

Urine Na^+^ excretion was measured with a flame photometer, while urine osmolality values were obtained via a micro-osmometer (Advanced Instruments, Norwood, MA). Urine Na^+^ concentration in the first series of experiments was measured with an automatic flame photometer (model IL943, Instrumentation Laboratory, Lexington, MA). The urine sodium concentration in follow up studies was measured with “EasyLyte Plus; Na/K/Cl” instrument from Medica Corp (Bedford, MA). Urine pH was measured with a pH meter (Accumet Basic AB15, Fisher Scientific, Hanover Park, IL).

### Blood composition and urine electrolytes analysis

Urine was collected under mineral oil. Serum and urine chloride concentration were measured using a digital chloridometer (HBI Haake Buchler Instruments, Inc.). Serum concentration of Na^+^, K^+^, Ca^++^, and HCO_3_^−^ were measured in the blood using i-STAT^R^-1 analyzer with i-STAT *EG7+* cartridges (Abbott Laboratories, Abbott Park, IL).

### Statistics analysis

The results for blood and urine chemistry data are presented as means ± SE. Statistical significance between experimental groups was determined by Student's unpaired *t-* test or Anova, and P<0.05 was considered significant. Unless indicated, 4–6 separate animals from each group were used for determination of expression levels (northern, western or immunofluorescence) and balanced studies.
